# Supplementary material for: MEIS-mediated suppression of human prostate cancer growth and metastasis through HOXB13-dependent regulation of proteoglycans
Source: eLife. 2020 Jun 18;9:e53600. doi: 10.7554/eLife.53600 (PMC7371429; doi:10.7554/eLife.53600)
Supplement: Supplementary file 7. [file elife-53600-supp7.docx]

**Supplemental File 7: KEY RESOURCES TABLE**

| **Reagent Type (species) or Resource** | **Designation** | **Source or Reference** | **Identifiers** | **Additional Information** |
| --- | --- | --- | --- | --- |
| antibody | Anti-c-Myc (Rabbit polyclonal) | Cell Signaling Technology | Cat# 9402, RRID:AB_2151827 | WB (1:1000) |
| antibody | Anti-Cas9 (7A9-3A3) (Mouse monoclonal) | Cell Signaling Technology | Cat# 14697, RRID:AB_2750916 | WB (1:1000) |
| antibody | Anti-Decorin Antibody (5E8E7) (Mouse monoclonal) | Abcam | Cat# ab181456 | WB (1:500) |
| antibody | Anti-HoxB13 (F-9) (Mouse monoclonal) | Santa Cruz Biotechnology | Cat# sc-28333, RRID:AB_627744 | WB (1:100)  PLA (1:100) |
| antibody | Anti-HOXB13 (EPR17371) (Rabbit polyclonal) | Abcam | Cat# ab201682 | Co-IP (2ug) |
| antibody | Anti-Smad2/3 XP(D7G7) (Rabbit polyclonal) | Cell Signaling Technology | Cat# 8685, RRID:AB_10889933 | WB (1:500) |
| antibody | Anti-MEIS1 - ChIP Grade (Rabbit polyclonal) | Abcam | Cat# ab19867, RRID:AB_776272 | ChIP (6ug)  PLA (1:1000) |
| antibody | Anti-MEIS1 (OTI2A3) (Mouse monoclonal) | OriGene Technologies | Cat# TA809619 | WB (1:1000) |
| antibody | Anti-MEIS2 (Rabbit polyclonal) | OriGene Technologies | Cat# TA337288 | WB (1:1000) |
| antibody | Anti-MEIS2 (MO1) (Mouse monoclonal) | Abnova | Cat# H000042120M01 | WB (1:1000) |
| antibody | IgG XP Isotype Control (DA1E) (Rabbit polyclonal) | Cell Signaling Technology | Cat# 3900, RRID:AB_1550038 | PLA (1:100) |
| antibody | IgG1 Isotype Control (G3A1) (Mouse monoclonal) | Cell Signaling Technology | at# 5415, RRID:AB_10829607 | PLA (1:1000) |
| antibody | Anti-p21 Waf1/Cip1 (12D1) (Rabbit polyclonal) | Cell Signaling Technology | Cat# 2947, RRID:AB_823586 | WB (1:500) |
| antibody | Anti-β-Actin (8H10D10) (Mouse monoclonal) | Cell Signaling Technology | Cat# 3700, RRID:AB_2242334 | WB (1:10,000) |
| antibody | Anti-c-MYC-pT58 (EPR17923) (Rabbit polyclonal) | Abcam | Cat# ab185655 | WB (1:500) |
| antibody | Anti-ITGB1 (Rabbit polyclonal) | Cell Signaling Technology | Cat# 4706, RRID:AB_823544 | WB (1:1000) |
| antibody | Anti-LUM (Rabbit polyclonal) | Thermo Fisher Scientific | Cat# PA5-14570, RRID:AB_2139479 | WB (1:300) |
| antibody | EGFR-pY1068 (D7A5) (Rabbit polyclonal) | Cell Signaling Technology | Cat# 3777, RRID:AB_2096270 | WB (1:1000) |
| antibody | EGFR (D38B1) (Rabbit polyclonal) | Cell Signaling Technology | Cat# 4267, RRID:AB_2246311 | WB (1:500) |
| antibody | SMAD2-pSer465/467 (138D4) (Rabbit polyclonal) | Cell Signaling Technology | Cat# 3108, RRID:AB_490941 | WB (1:200) |
| antibody | TGFBR3 (D11G10) (Rabbit polyclonal) | Cell Signaling Technology | Cat# 5544, RRID:AB_10698740 | WB (1:200) |
| antibody | IRDye® 680LT anti-Mouse IgG (H + L) (Goat polyclonal) | Licor | Cat# P/N 925-68020, RRID:AB_2687826 | WB (1:10,000) |
| antibody | IRDye® 800CW anti-Rabbit IgG (H + L) (Goat polyclonal) | Licor | Cat# 925-32211, RRID:AB_2651127 | WB (1:10,000) |
| recombinant DNA reagent | LV105-MEIS1  (plasmid) | GeneCopoeia | Custom order | Puromycin-resistance |
| recombinant DNA reagent | LV105-MEIS2A  (plasmid) | GeneCopoeia | Custom order | Puromycin-resistance |
| recombinant DNA reagent | LV105-MEIS2D  (plasmid) | GeneCopoeia | Custom order | Puromycin-resistance |
| recombinant DNA reagent | LV105-MEIS2E  (plasmid) | GeneCopoeia | Custom order | Puromycin-resistance |
| chemical compound, drug | (+)-Aphidicolin, proliferation inhibitor | Cayman Chemical Company | Cat# 14007 |  |
| chemical compound, drug | METRIBOLONE (R1881) >99% | Fisher Scientific | Cat# 50152311 |  |
| chemical compound, drug | D-Luciferin Firefly, sodium salt monohydrate | Goldbio | Cat# LUCNA-2G |  |
| commercial assay or kit | iDeal ChIP-seq kit for Transcription Factors | Diagenode | Cat # C01010055 |  |
| commercial assay or kit | CyQUANT Direct Cell Proliferation Assay | Thermo Fisher Scientific | Cat# C35011 |  |
| commercial assay or kit | Duolink® In Situ Red Starter Kit Mouse/Rabbit | Sigma-Aldrich | Cat# DUO92101 |  |
| commercial assay or kit | KAPA Stranded mRNA-Seq Kit | Kapa Biosystems | Cat# KK8420 |  |
| commercial assay or kit | Library Quantification Kit – Illumina/Universal | Kapa Biosystems | Cat# KK4824 |  |
| commercial assay or kit | Low throughput Library prep kit | Kapa Biosystems | Cat# KK8230 |  |
| commercial assay or kit | Corning® Transwell® polycarbonate membrane cell culture inserts, 8 um pore size. | Corning | Cat# CLS3422 |  |
| commercial assay or kit | Qubit® dsDNA HS Assay Kit | Thermo Fisher Scientific | Cat# Q32851 |  |
| commercial assay or kit | siLentFect Lipid Reagent for RNAi | Bio-Rad | Cat# 1703360 |  |
| commercial assay or kit | ViraPower Lentiviral Packaging Mix | Thermo Fisher Scientific | Cat# K497500 |  |
| commercial assay or kit | cOmplete™ Mini Protease Inhibitor Cocktail | Sigma-Aldrich | Cat# 11873580001 |  |
| commercial assay or kit | ViaStain™ PI Cell Cycle Kit | Nexcelom Bioscience | Cat# CSK-0112 |  |
| commercial assay or kit | Click-iT™ TUNEL Alexa Fluor™ 647 Imaging Assay, for microscopy & HCS | Thermo Fisher Scientific | Cat# C10247 |  |
| Other | Raw and analyzed sequencing data | This paper | GSE132717 | Deposited data |
| Other | Human reference cDNA ENSEMBL release 93, GRCh38.p12 | Genome Reference Consortium Human Build 38 | https://useast.ensembl.org/Homo_sapiens/Info/Index | Deposited data |
| Other | Human reference genome ENSEMBL release 93, GRCh38.p12 | Genome Reference Consortium Human Build 38 | https://useast.ensembl.org/Homo_sapiens/Info/Index | Deposited data |
| Other | Human reference ncRNA ENSEMBL release 93, GRCh38.p12 | Genome Reference Consortium Human Build 38 | https://useast.ensembl.org/Homo_sapiens/Info/Index | Deposited data |
| cell line (*Homo-sapiens*) | CWR22Rv1 (human; male) | American Type Culture Collection | Cat# CRL-2505, RRID:CVCL_1045 |  |
| cell line (*Homo-sapiens*) | HEK293T (human; fetus) | American Type Culture Collection | Cat# CRL-3216, RRID:CVCL_0063 |  |
| cell line (*Homo-sapiens*) | PrEC 9266 short-term culture (human; male) | This Paper | Chen et al., CCR 2012  (PMID 22723371) | Short-term primary culture of prostate epithelial cells |
| cell line (*Homo-sapiens*) | PrEC 5061 short-term culture (human; male) | This Paper | Chen et al., CCR 2012  (PMID 22723371) | Short-term primary culture of prostate epithelial cells |
| cell line (*Homo-sapiens*) | PrEC 7510 short-term culture (human; male) | This Paper | Chen et al., CCR 2012  (PMID 22723371) | Short-term primary culture of prostate epithelial cells |
| cell line (*Homo-sapiens*) | LAPC-4 (human; male) | American Type Culture Collection | Cat# CRL-13009, RRID:CVCL_4744 |  |
| Strain, strain background (*M. musculus*) | Mouse: Hsd:Athymic Nude-*Foxn1^nu^* male | Envigo (previously Harlan) |  | For in vivo xenograft metastasis assays |
| sequenced-based reagent | Alt-R™ CRISPR-Cas9 tracrRNA, ATTO™ 550 | Integrated DNA Technologies | Cat# 1075927 |  |
| sequenced-based reagent | Custom Alt-R™ CRISPR-Cas9 crRNA: HOXB13 crRNA | Integrated DNA Technologies, This paper | PCR Primers | TTGACAGCAGGCATCAGCGT |
| sequenced-based reagent | Primer: MEIS1 forward: | Integrated DNA Technologies, This paper | PCR Primers | TGGCTGTTCCAGCATCTAACACAC |
| sequenced-based reagent | Primer: MEIS1 reverse: | Integrated DNA Technologies, This paper | PCR Primers | ACTGGTCTATCATGGGCTGCAC |
| sequenced-based reagent | Primer: pan-MEIS2 forward: | Integrated DNA Technologies, This paper | PCR Primers | ATCTCGCTGACCATAACCCT |
| sequenced-based reagent | Primer: pan-MEIS2 reverse: | Integrated DNA Technologies, This paper | PCR Primers | CCGGATCATCATCGTCACCT |
| sequenced-based reagent | Primer: MEIS2E specific forward: | Integrated DNA Technologies, This paper | PCR Primers | GGAGACAACAGCAGTGAGCA |
| sequenced-based reagent | Primer: MEIS2E specific reverse: | Integrated DNA Technologies, This paper | PCR Primers | TTCTTCTGGCATTAATAAACTGTGA |
| sequenced-based reagent | Primer: DCN forward: | Integrated DNA technologies, (Kim et al., 2014) | PCR Primers | ATGAAGGCCACTATCATCCTCC |
| sequenced-based reagent | Primer: DCN reverse: | Integrated DNA technologies, (Kim et al., 2014) | PCR Primers | GTCGCGGTCATCAGGAACTT |
| sequenced-based reagent | Primer: RPL13A forward: | Integrated DNA Technologies, This paper | PCR Primers | GGAGCAAGGAAAGGGTCTTAG |
| sequenced-based reagent | Primer: RPL13A reverse: | Integrated DNA Technologies, This paper | PCR Primers | GGTTGCTCTTCCTATTGGTCATA |
| sequenced-based reagent | Primer: DCN Genomic #1 Forward: | Integrated DNA Technologies, This paper | PCR Primers | GCACACGTGTTTGGTATTCTCA |
| sequenced-based reagent | Primer: DCN Genomic #1 Reverse: | Integrated DNA Technologies, This paper | PCR Primers | CCTGATGCCTGATGAAGAGTGA |
| sequenced-based reagent | Primer: DCN Genomic #2 Forward: | Integrated DNA Technologies, This paper | PCR Primers | ATTGGGACATTGGTGGTGTACT |
| sequenced-based reagent | Primer: DCN Genomic #2 Reverse: | Integrated DNA Technologies, This paper | PCR Primers | ATGCTTGCCTTGAGCAGAAAAG |
| sequenced-based reagent | Primer: LUM Genomic Forward: | Integrated DNA Technologies, This paper | PCR Primers | CAGTTAGGTCTGCTCCCCAC |
| sequenced-based reagent | Primer: LUM Genomic Reverse: | Integrated DNA Technologies, This paper | PCR Primers | CTCCCTTCATTCATTGTTCATCCA |
| sequenced-based reagent | Primer: TGFBR1 Genomic Forward: | Integrated DNA Technologies, This paper | PCR Primers | CCAGCTAGAGTGCGGGATG |
| sequenced-based reagent | Primer: TGFBR1 Genomic Reverse: | Integrated DNA Technologies, This paper | PCR Primers | TCCCACAAGCGCACATTTCT |
| sequenced-based reagent | Human Negative Control ChIP Primer Set 3 | Active Motif | Cat# 71023 |  |
| sequenced-based reagent | Human Positive Control ChIP Primer Set ACTB-1 | Active Motif | Cat# 71003 |  |
| sequenced-based reagent | ON-TARGETplus Human DCN (1634) siRNA – SMARTpool | Dharmacon | Cat# L-021491-00-0005 |  |
| sequenced-based reagent | ON-TARGETplus Non-targeting Control pool siRNA | Dharmacon | Cat# D-001810-10-05 |  |
| recombinant DNA reagent | pT2-EF1a-Cas9-P2A-puro | The Lab of Dr. Brendan Looyenga. This paper. | N/A |  |
| recombinant DNA reagent | pCMV(CAT)T7-SB100 | Addgene | Cat# 34879, RRID:Addgene_34879 | (Mátés et al., 2009) |
| software, algorithm | Bioconductor 3.0 | N/A | [https://www.bioconductor.org](https://www.bioconductor.org/) |  |
| software, algorithm | Bowtie2 | (Langmead and Salzberg, 2012) | http://bowtie-bio.sourceforge.net/bowtie2/index.shtml |  |
| software, algorithm | CHOPCHOP | (Labun et al., 2016) | http://chopchop.cbu.uib.no/ |  |
| software, algorithm | Enrichr | (Chen et al., 2013) | http://amp.pharm.mssm.edu/Enrichr/ |  |
| software, algorithm | FastQC | Babraham Bioinformatics | <http://www.bioinformatics.babraham.ac.uk/projects/fastqc/> |  |
| software, algorithm | Fiji | (Schindelin et al., 2012) | http://imagej.net/Fiji |  |
| software, algorithm | GSEA 3.0 | Broad Institute | http://software.broadinstitute.org/gsea/index.jsp |  |
| software, algorithm | HOMER v4.9.1 | (Heinz et al., 2010) | http://homer.ucsd.edu/homer/index.html |  |
| software, algorithm | Kallisto v0.43.1 | (Bray et al., 2016) | https://pachterlab.github.io/kallisto/download |  |
| software, algorithm | MACS2 v2.1.2 | (Zhang et al., 2008) | https://github.com/taoliu/MACS |  |
| software, algorithm | Picard Tools v2.18.10 | Broad Institute | https://broadinstitute.github.io/picard/ |  |
| software, algorithm | R | The R Foundation | [https://www.r-project.org](https://www.r-project.org/) |  |
| software, algorithm | R package edgeR v3.22.3 | (Robinson et al., 2010) | <https://bioconductor.org/packages/release/bioc/html/edgeR.html> |  |
| software, algorithm | R package tximport v1.8.0 | (Soneson et al., 2015) | https://bioconductor.org/packages/release/bioc/html/tximport.html |  |
| software, algorithm | R package survival v2.43-3 | (Therneau, 2015) | https://github.com/therneau/survival |  |
| software, algorithm | Samtools v1.6 | (Li et al., 2009) | http://samtools.sourceforge.net/ |  |
| software, algorithm | Trimmomatic v0.38 | (Bolger et al., 2014) | <http://www.usadellab.org/cms/index.php?page=trimmomatic> |  |
| software, algorithm | STAMP | (Mahony and Benos, 2007) | http://www.benoslab.pitt.edu/stamp/ |  |
| software, algorithm | FCS Express Cytometry 6 | De Novo Software | https://www.denovosoftware.com/ |  |
| software, algorithm | MEME Suite v5.0.5 | (Bailey et al., 2009) | http://meme-suite.org/index.html |  |
| software, algorithm | deepTools v3.2.0 | (Ramírez et al., 2016) | https://deeptools.readthedocs.io/en/develop/index.html |  |
